# Traditional Medicinal Plant *Dahlia pinnata* Selectively Suppresses TNF-α Expression Through Modulation of NF-κB and p38 Signaling

**DOI:** 10.3390/ijms27021122

**Published:** 2026-01-22

**Authors:** HyeRin Woo, Yeji Lee, Jongmin Ahn, Yongxin Jin, Weihui Wu, Un-Hwan Ha

**Affiliations:** 1 Department of Biotechnology and Bioinformatics, Korea University, Sejong 30019, Republic of Korea; gpfls99017@korea.ac.kr (H.W.); yejee90@korea.ac.kr (Y.L.); 2 Interdisciplinary Graduate Program for Artificial Intelligence Smart Convergence Technology, Korea University, Sejong 30019, Republic of Korea; 3 Natural Product Research Center, Korea Research Institute of Bioscience & Biotechnology, Cheongju 28116, Republic of Korea; jmahn@kribb.re.kr; 4 State Key Laboratory of Medicinal Chemical Biology, Key Laboratory of Molecular Microbiology and Technology of the Ministry of Education, Department of Microbiology, Nankai University, Tianjin 300071, China; yxjin@nankai.edu.cn (Y.J.); wuweihui@nankai.edu.cn (W.W.)

**Keywords:** *Dahlia pinnata*, fractionation, NF-κB, LPS, p38, *Pseudomonas aeruginosa*, TNF-α

## Abstract

Tumor necrosis factor-α (TNF-α) is a central mediator of inflammatory pathology; thus, the selective suppression of TNF-α without causing broad immunosuppression remains a critical therapeutic goal. This study investigated the anti-inflammatory potential and underlying mechanisms of *Dahlia pinnata* (*D. pinnata*) extract in human monocytes and epithelial cells. We demonstrate that *D. pinnata* extract selectively suppresses basal TNF-α expression in THP-1 monocytes and BEAS-2B bronchial epithelial cells, with minimal impact on IL-1β, IL-6, or IL-10 and without inducing cytotoxicity. The extract also potently attenuated TNF-α induction triggered by *Pseudomonas aeruginosa* infection or lipopolysaccharide (LPS) stimulation. Notably, *D. pinnata* extract exhibited stronger and broader TNF-α-suppressive effects than dexamethasone, particularly in monocytes where dexamethasone was ineffective under the tested conditions. Mechanistic analyses revealed that the extract suppresses TNF-α expression primarily through the inhibition of NF-κB signaling, accompanied by enhanced p38 MAPK activation. Fractionation of the extract identified two active fractions (06 and 07) that robustly suppressed TNF-α expression under both basal and stimulated conditions while maintaining low cytotoxicity. These fractions recapitulated the signaling profile of the crude extract by inhibiting NF-κB activation and promoting p38 signaling. Collectively, our findings identify *D. pinnata* as a rich source of bioactive compounds that selectively suppresses TNF-α through the coordinated modulation of NF-κB and p38 pathways, highlighting its potential as a scaffold for developing targeted anti-inflammatory therapeutics.

## 1. Introduction

Activation of the innate immune system is essential for maintaining physiological homeostasis during infection or trauma [1,2]. Innate immune responses are characterized by the temporal regulation of inflammatory cytokines—including IL-1β, IL-6, IL-10, and TNF-α—produced by diverse immune cell types [3,4]. Monocytes, in particular, play a central role as they are recruited to infection sites and differentiate into macrophages to orchestrate these responses [5]. Impaired monocyte function is therefore associated with increased susceptibility to infection [6,7]. As integral components of innate immunity, monocytes support pathogen defense, regulate inflammation, preserve tissue integrity, and contribute to metabolic homeostasis. Conversely, excessive monocyte activation promotes inflammatory, autoimmune, and infectious diseases, driven by the overproduction of pro-inflammatory cytokines that induce tissue damage, systemic inflammation, and immune dysregulation [8,9].

TNF-α, originally identified for its tumoricidal activity [10], is a pleiotropic cytokine essential for normal inflammatory and immune functions; however, it is also central to the pathogenesis of various autoimmune and inflammatory disorders [11,12,13,14,15]. In rheumatoid arthritis (RA), for example, elevated TNF-α drives immune-cell recruitment and stimulates the production of other inflammatory mediators, including matrix metalloproteinases, which lead to cartilage and bone destruction [16,17,18,19,20]. Elevated TNF-α also contributes to the pathophysiology of psoriasis and inflammatory bowel disease (IBD), as well as insulin resistance in type 2 diabetes [21,22,23]. Notably, TNF-α is a primary mediator in the development of *Pseudomonas aeruginosa* (*P. aeruginosa*)-induced septic shock [24]. Accordingly, therapeutic strategies that neutralize TNF-α or inhibit its expression and signaling have proven effective in ameliorating RA and Crohn’s disease [11,14]. This has established TNF-α blockade as a cornerstone approach for treating immune-mediated disorders, delivering substantial clinical impact [25,26,27,28,29].

Plants serve as rich sources of structurally diverse natural products with broad biological activities, including potent anti-inflammatory effects [30]. However, their clinical translation is frequently hindered because many of these complex molecules are difficult to synthesize or produce at scale. Consequently, considerable effort has focused on identifying natural compounds with anti-inflammatory activity and optimizing their structures to enhance efficacy while reducing toxicity. For example, triptolide (TP)—a bioactive constituent of *Tripterygium wilfordii* with robust anti-inflammatory and immunosuppressive properties—suppresses TNF-α expression while concurrently enhancing IL-10 production [31,32,33]. Despite this potential, the clinical application of TP in RA is constrained by significant toxicity, a narrow therapeutic window, and poor bioavailability, underscoring the critical need for safer alternatives.

*D. pinnata*, an herbaceous member of the *Asteraceae* family native to Mexico, has long been valued as both an ornamental and medicinal plant. Ethnobotanical records document its traditional use among indigenous populations as a diuretic, febrifuge, and antitussive, as well as a remedy for colic and inflammation [34]. Furthermore, its tubers are rich in inulin and have been utilized as a dietary substitute for diabetic patients and for general detoxification [34,35]. Recent phytochemical investigations have identified a range of bioactive constituents—including flavonoids, terpenoids, phenolic acids, and anthocyanins—which are commonly associated with anti-inflammatory, antioxidant, and antimicrobial activities [36]. Key compounds identified include kaempferol-3-rutinoside, pelargonidin-3-(6″-malonylglucoside)-5-glucoside, rutin, kaempferol-3-(2″,3″-diacetyl-4″-p-coumaroylrhamnoside), and myricetin-3-(2″-galloylrhamnoside). These ethnomedicinal applications, particularly for fever, cough, and colic, are physiologically linked to inflammatory processes involving cytokines such as TNF-α. However, despite its established traditional use, the molecular mechanisms underlying the anti-inflammatory actions of *D. pinnata* remain largely unexplored. 

Motivated by the growing interest in plant-derived modulators of immune signaling, we investigated whether *D. pinnata* extracts attenuate pro-inflammatory cytokine expression, with a specific focus on TNF-α. Bioassay-guided fractionation identified specific fractions that recapitulated these anti-inflammatory effects. Mechanistic studies further implicated the NF-κB and p38 MAPK pathways—key regulators of inflammatory signaling—in mediating these responses. Together, these findings highlight the immunopharmacological potential of *D. pinnata* and support its development as a novel plant-derived modulator of inflammation and innate immunity.

## 2. Results

### 2.1. D. pinnata Extract Selectively Suppresses TNF-α Expression Under Basal Conditions

**Figure 1:**
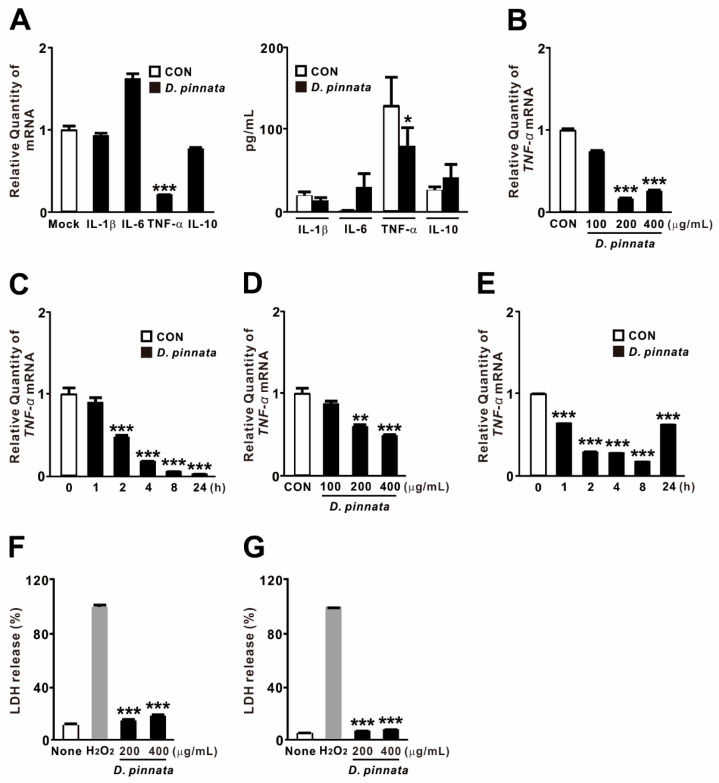
*Dahlia pinnata* (*D. pinnata*) extract selectively suppresses TNF-α expression under basal conditions. THP-1 cells (**A**–**C**,**F**) and BEAS-2B cells (**D**,**E**,**G**) were treated with 200 μg/mL of *D. pinnata* extract (**A**,**C**,**E**) or with the indicated concentrations (**B**,**D**,**F**,**G**) for 4 h. Following treatment, mRNA and protein levels were quantified by qRT-PCR and ELISA, and cytotoxicity was assessed by LDH release assay. Data are presented as mean ± SD (n = 3). * *p* < 0.05, ** *p* < 0.01, *** *p* < 0.001 vs. control (CON; (**A**–**E**)) or H_2_O_2_-treated cells (**F**,**G**). Dimethyl sulfoxide (DMSO) was used as vehicle control (CON or None).

To investigate the effects of *D. pinnata* on inflammatory cytokine regulation, THP-1 monocytes were treated with the extract. As shown in Figure 1A, *D. pinnata* extract markedly reduced the expression of TNF-α at both mRNA and protein levels while having minimal effects on other pro-inflammatory cytokines (IL-1β and IL-6) and the anti-inflammatory cytokine IL-10. This suppression of TNF-α was both dose- and time-dependent (Figure 1B,C), with the most pronounced inhibition observed at 200 μg/mL and maximal reduction achieved at 24 h. Consistently, *D. pinnata* extract also reduced TNF-α expression in BEAS-2B bronchial epithelial cells, although the magnitude and kinetics differed slightly from those observed in monocytes (Figure 1D,E). To exclude cytotoxicity as a confounding factor, lactate dehydrogenase (LDH) release was measured in both cell types. The extract resulted in significantly lower LDH release compared to the hydrogen peroxide positive control (Figure 1F,G), confirming its low cytotoxicity. Collectively, these results suggest that *D. pinnata* extract selectively suppresses TNF-α expression under basal conditions and that this effect is not attributable to impaired cell viability.

### 2.2. D. pinnata Extract Suppresses P. aeruginosa-Induced TNF-α Expression

**Figure 2:**
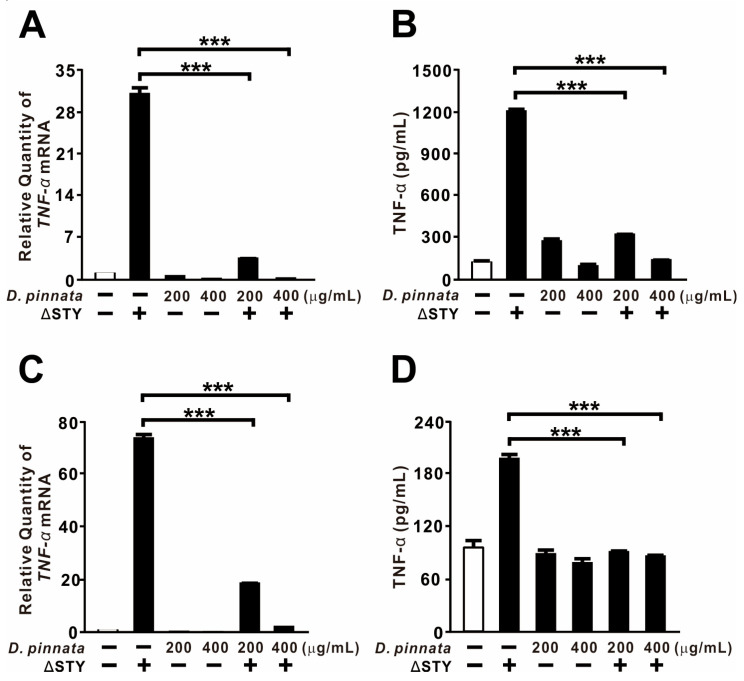
*D. pinnata* extract suppresses *P. aeruginosa*-induced TNF-α expression. THP-1 cells (**A**,**B**) and BEAS-2B cells (**C**,**D**) were pretreated with *D. pinnata* extract (200 or 400 μg/mL) for 1 h, followed by infection with PAKΔSTY (ΔSTY) at a multiplicity of infection (MOI) of 2 for 4 h. TNF-α mRNA levels were measured by qRT-PCR, and TNF-α protein levels were determined by ELISA. Data are presented as mean ± SD (n = 3). *** *p* < 0.001.

We next examined whether *D. pinnata* extract could suppress TNF-α expression induced by bacterial infection, a potent trigger of inflammatory signaling. As expected, infection with the *P. aeruginosa* PAKΔSTY mutant strain—which lacks T3SS effectors—strongly upregulated TNF-α expression at both the mRNA and protein levels in THP-1 monocytes (Figure 2A,B). This is consistent with previous reports identifying *P. aeruginosa* as a robust inducer of TNF-α in human monocytes [37]. Pretreatment with *D. pinnata* extract significantly attenuated this induction in a dose-dependent manner. Similar inhibitory effects were observed in BEAS-2B epithelial cells, albeit with minor differences in magnitude (Figure 2C,D). Collectively, these findings demonstrate that *D. pinnata* extract effectively suppresses infection-induced TNF-α expression across different cell types.

### 2.3. D. pinnata Extract Exerts Stronger Suppressive Effects than Dexamethasone

**Figure 3:**
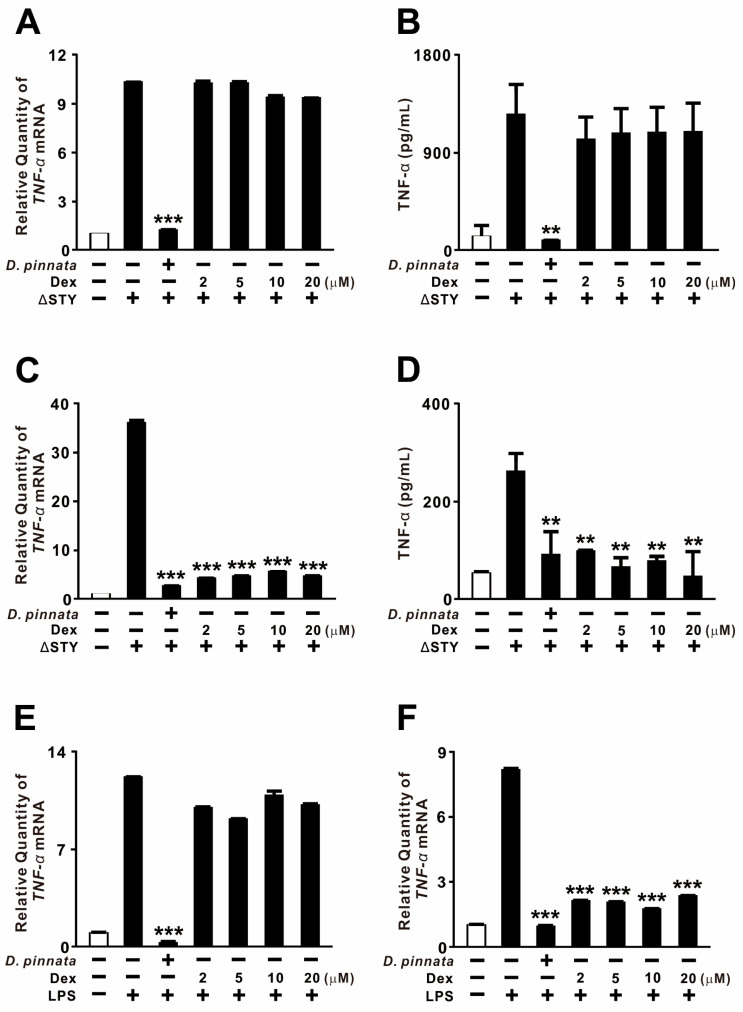
*D. pinnata* extract exerts stronger suppressive effects than dexamethasone. THP-1 cells (**A**,**B**,**E**) and BEAS-2B cells (**C**,**D**,**F**) were pretreated with *D. pinnata* extract (200 μg/mL) or dexamethasone (Dex; 2, 5, 10, or 20 μM) for 1 h, followed by infection with PAKΔSTY (MOI 2; (**A**–**D**)) or stimulation with LPS (0.5 μg/mL; (**E**,**F**)) for 4 h. TNF-α mRNA levels were quantified by qRT-PCR, and TNF-α protein levels were measured by ELISA. Data are expressed as mean ± SD (n = 3). ** *p* < 0.01, *** *p* < 0.001 vs. PAKΔSTY or LPS alone.

Dexamethasone is a well-established suppressor of TNF-α release from human monocytes, which represent a critical therapeutic target in inflammatory disease [38,39]. We therefore compared the suppressive efficacy of *D. pinnata* extract with that of dexamethasone. In THP-1 monocytes, pretreatment with *D. pinnata* extract significantly reduced *P. aeruginosa* PAKΔSTY-induced TNF-α expression at both the mRNA and protein levels, whereas dexamethasone exhibited no inhibitory effect (Figure 3A,B). In contrast, both treatments successfully suppressed TNF-α expression in BEAS-2B epithelial cells (Figure 3C,D). When *P. aeruginosa*–derived LPS was employed as the stimulus, *D. pinnata* extract consistently reduced TNF-α levels in both cell types, while dexamethasone again failed to inhibit TNF-α expression in monocytes (Figure 3E,F). These findings demonstrate that *D. pinnata* extract exerts more potent and broader TNF-α–suppressive effects than dexamethasone under these experimental conditions, particularly within the monocytic lineage.

### 2.4. D. pinnata Extract Reduces AKT Activation

**Figure 4:**
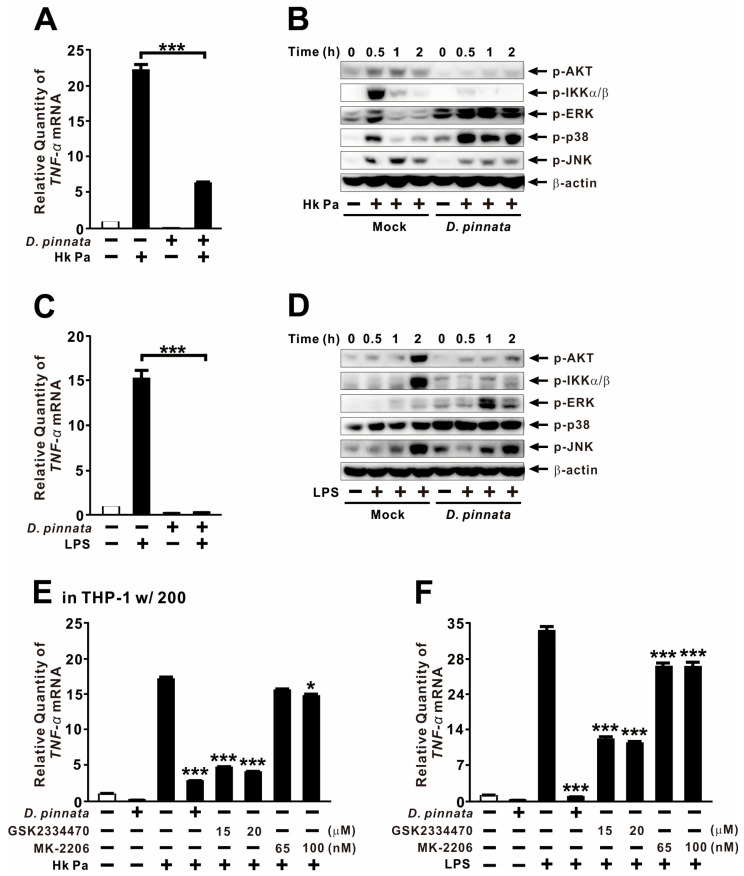
*D. pinnata* extract reduces AKT activation. THP-1 cells were pretreated with either *D. pinnata* extract (200 μg/mL) or chemical inhibitors (**E**,**F**) for 1 h, followed by stimulation with heat-killed *P. aeruginosa* PAK strain (Hk Pa; MOI 5; (**A**,**B**,**E**)) or LPS (0.5 μg/mL; (**C**,**D**,**F**)) for 4 h (**A**,**C**,**E**,**F**) or indicated times (**B**,**D**). TNF-α mRNA levels were quantified by qRT-PCR, TNF-α protein levels were measured by ELISA, and phosphorylation levels were assessed by immunoblot analysis. Data are presented as mean ± SD (n = 3). Results in (**B**,**D**) are representative of three independent experiments. * *p* < 0.05, *** *p* < 0.001 vs. Hk Pa or LPS alone. Inhibitors: GSK2334470 (PDK1), MK-2206 (AKT).

Because *P. aeruginosa* LPS potently induces TNF-α expression, we stimulated THP-1 cells with either heat-killed *P. aeruginosa* (HK-Pa) or purified LPS to investigate the signaling pathways targeted by *D. pinnata*. Pretreatment with the extract significantly reduced HK-Pa–induced TNF-α expression (Figure 4A), confirming its ability to suppress agonist-mediated cytokine induction. Given that the AKT, NF-κB, and MAPK pathways are well-established regulators of TNF-α in immune cells responding to microbial products [40,41,42], we next examined their involvement. Mechanistic analysis revealed that *D. pinnata* markedly inhibited the phosphorylation of AKT and IKKα/β, while slightly decreasing JNK phosphorylation and—interestingly—enhancing ERK and p38 phosphorylation (Figure 4B).

Under purified LPS stimulation, the suppressive effects of *D. pinnata* extract on TNF-α were more pronounced than those observed with HK-Pa, which contains a diverse array of outer-membrane agonists; this suggests a potential selective suppression of LPS-driven signaling (Figure 4C). Although LPS induced a distinct activation pattern among signaling mediators, *D. pinnata* consistently suppressed AKT and IKKα/β while enhancing ERK and p38 phosphorylation, with negligible effects on JNK (Figure 4D). Because PDK1 is a critical upstream activator of AKT, we further evaluated its role in TNF-α regulation. Pretreatment with the PDK1 inhibitor GSK2334470 reduced HK-Pa–induced TNF-α expression to an extent similar to that of *D. pinnata*, whereas the AKT inhibitor MK-2206 had little effect (Figure 4E). In the LPS model, the AKT inhibitor yielded only minor reductions, while the PDK1 inhibitor exerted clear but weaker effects compared with *D. pinnata* (Figure 4F). Collectively, these results indicate that *D. pinnata* extract suppresses TNF-α expression, at least in part, through the inhibition of AKT signaling.

### 2.5. Suppression of TNF-α Expression by D. pinnata Extract Is Mediated Through Modulation of NF-κB and p38 Signaling

**Figure 5:**
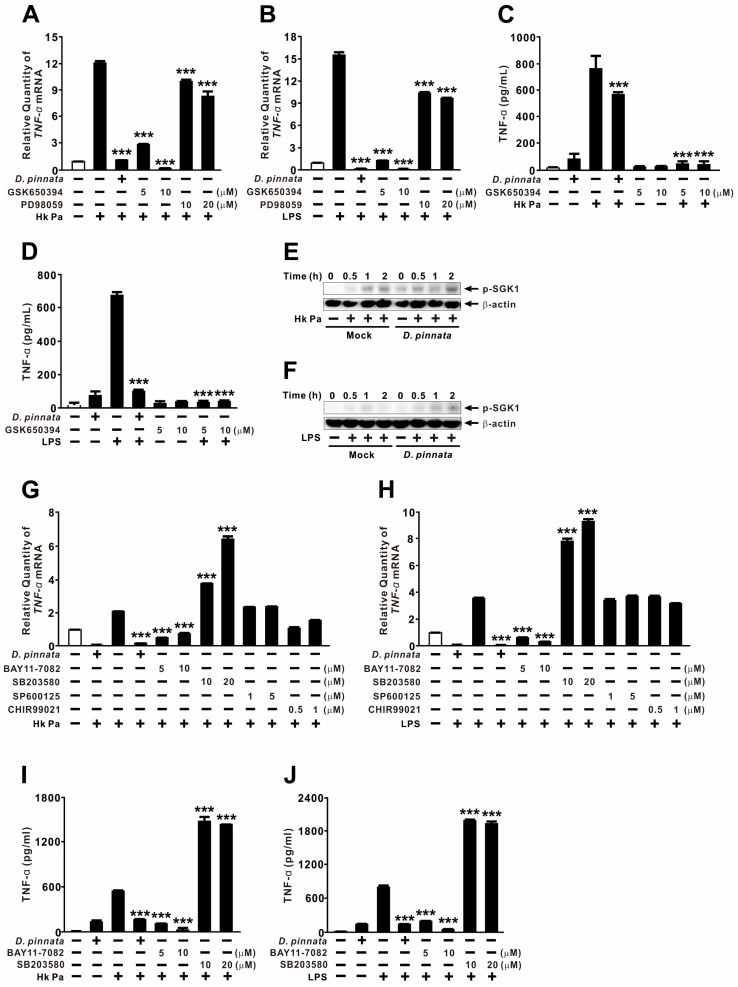
Suppression of TNF-α expression by *D. pinnata* extract is mediated through modulation of NF-κB and p38 signaling. THP-1 cells were pretreated with either *D. pinnata* extract (200 μg/mL) or chemical inhibitors (**A**–**D**,**G**–**J**) for 1 h, followed by stimulation with heat-killed *P. aeruginosa* PAK strain (Hk Pa; MOI 5 for (**A**,**C**,**E**,**G**); MOI 1 for (**I**)) or LPS (0.5 μg/mL for (**B**,**D**,**F**,**H**); 0.1 μg/mL for (**J**)) for 4 h (**A**–**D**,**G**–**J**) or indicated times (**E**,**F**). TNF-α mRNA levels were quantified by qRT-PCR, TNF-α protein levels were measured by ELISA, and phosphorylation levels were assessed by immunoblot analysis. Data are presented as mean ± SD (n = 3). Results in (**E**,**F**) are representative of three independent experiments. *** *p* < 0.001 vs. Hk Pa or LPS alone. Inhibitors: GSK650394 (SGK1), PD98059 (ERK), BAY 11-7082 (NF-κB), SB203580 (p38), SP600125 (JNK), and CHIR99021 (GSK3β).

Since the inhibition of PDK1 reduced TNF-α expression while AKT inhibition yielded only minor effects, we investigated the potential role of SGK1, which also requires PDK1 for canonical activation [43]. Pretreatment with an SGK1 inhibitor significantly reduced TNF-α expression induced by HK-Pa or purified LPS in a dose-dependent manner, reaching levels comparable to those achieved by the *D. pinnata* extract (Figure 5A,B). In contrast, the ERK inhibitor PD98059 produced only minor effects, despite the extract clearly enhancing ERK phosphorylation (Figure 4B,D); this suggests that ERK activation is unlikely to mediate the extract-induced suppression of TNF-α. The potential involvement of SGK1 was further evaluated through the measurement of TNF-α protein levels, which showed a similar reduction (Figure 5C,D). However, the extract did not decrease SGK1 activation and instead slightly increased its phosphorylation (Figure 5E,F). These results indicate that while SGK1 activity is necessary for LPS-induced TNF-α production, it is not the direct target of *D. pinnata*-mediated suppression.

Because the extract decreased IKKα/β phosphorylation but enhanced p38 phosphorylation (Figure 4B,D), we next investigated their respective contributions to cytokine regulation. We also evaluated the JNK inhibitor SP600125 and the GSK3β inhibitor CHIR99021, as JNK activation was slightly attenuated by the extract, and GSK3β is known to modulate NF-κB activation [44,45]. Pretreatment with the NF-κB inhibitor BAY 11-7082 suppressed HK-Pa- or LPS-induced TNF-α expression to a degree similar to that of the extract. In contrast, the p38 inhibitor SB203580 unexpectedly enhanced TNF-α expression (Figure 5G,H). These effects were further confirmed at the protein level (Figure 5I,J). While JNK and GSK3β inhibition had minimal impact, the collective findings suggest that *D. pinnata* extract suppresses TNF-α expression primarily through the inhibition of NF-κB signaling and the concomitant activation of p38 signaling, the latter of which may serve as a negative feedback regulator in this context.

### 2.6. Fractions 06 and 07 of D. pinnata Extract Potently Suppress TNF-α Expression

**Figure 6:**
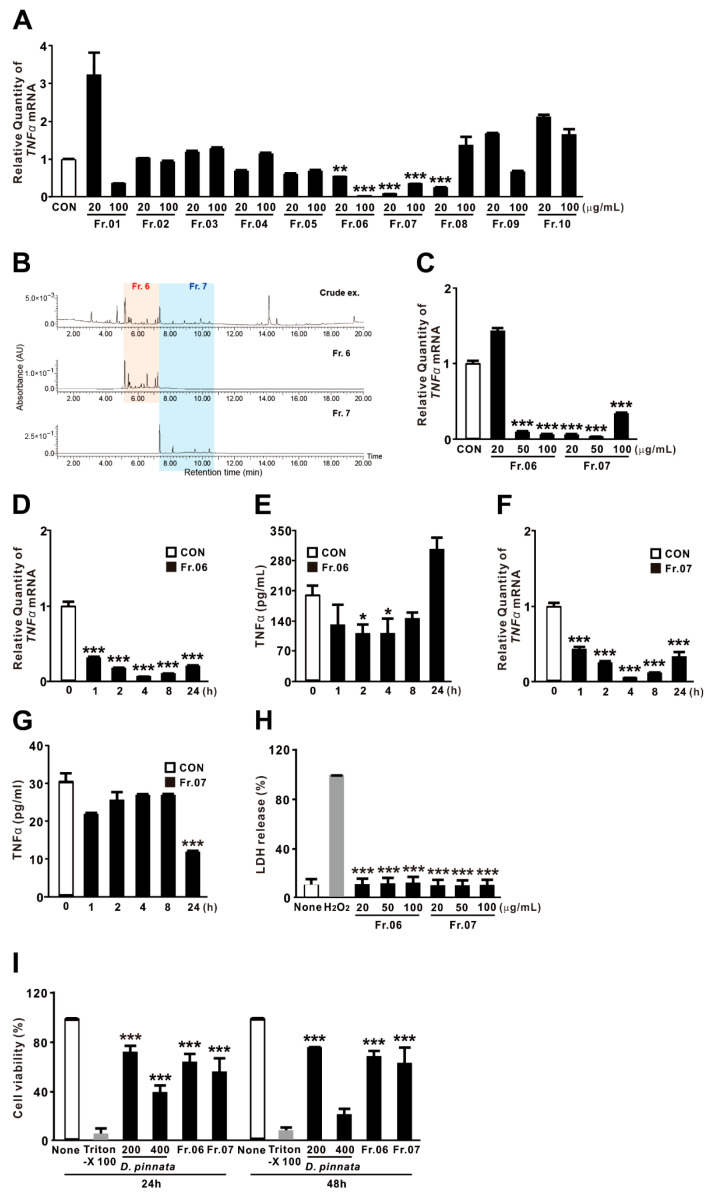
Fractions 06 and 07 of *D. pinnata* extract potently suppress TNF-α expression. (**A**,**C**) THP-1 cells were treated with various fractions of *D. pinnata* extract at the indicated concentrations for 4 h. (**B**) Representative HPLC chromatograms of Fr.06 and Fr.07 showing distinct peaks at the indicated retention times. (**D**–**G**) THP-1 cells were treated with Fr.06 (50 μg/mL) or Fr.07 (20 μg/mL) for the indicated times. (**H**) THP-1 cells were treated with Fr.06 or Fr.07 at the indicated concentrations for 4 h. (**I**) THP-1 cells were treated with *D. pinnata* extract (200 or 400 μg/mL), Fr.06 (50 μg/mL), or Fr.07 (20 μg/mL) for 24 h or 48 h. Following treatment, TNF-α mRNA levels were quantified by qRT-PCR, and TNF-α protein levels were determined by ELISA. Cytotoxicity was assessed by LDH release assay or Alamar Blue assay. Data are presented as mean ± SD (n = 3). * *p* < 0.05, ** *p* < 0.01, *** *p* < 0.001 vs. control (CON; (**A**,**C**–**G**)), H_2_O_2_-treated cells (**H**), or Triton X-100-treated cells (**I**). DMSO was used as vehicle control (CON or None).

The composition of *D. pinnata* extract was analyzed by UV chromatography at 280 nm to tentatively identify constituent peaks, yielding 10 fractions as described in Section 4. The UPLC-UV analysis data and the tentative identification of flavonoids are provided in the Supplementary Materials (File S1 and File S2, respectively). We next examined the inhibitory effects of each fraction on TNF-α expression. As shown in Figure 6A, fractions 06 and 07 markedly reduced TNF-α expression at both tested doses, suggesting that they contain bioactive compounds with potent suppressive activity. The UV chromatogram indicated that the peaks corresponding to these fractions eluted between 5.0 and 7.3 min for fraction 06 and 7.3–10.5 min for fraction 07 (Figure 6B). Based on these findings, fractions 06 and 07 were selected for further investigation, whereas fractions 01 and 08 were excluded because they exhibited inhibitory effects only at a single dose. The suppressive effects of fractions 06 and 07 were further confirmed in a dose-dependent manner (Figure 6C), and concentrations of 50 μg/mL for fraction 06 and 20 μg/mL for fraction 07 were selected for subsequent experiments. We then evaluated their inhibitory effects over time. As shown in Figure 6D,F, both fractions significantly reduced TNF-α mRNA expression under basal conditions in a time-dependent manner, with maximal suppression observed at 4 h post-treatment. At the protein level, fraction 06 exhibited only modest inhibitory activity, whereas fraction 07 induced maximal suppression at 24 h (Figure 6E,G).

To rule out the possibility that TNF-α suppression was due to cellular damage, we measured LDH release. Both fractions induced minimal LDH release compared with the hydrogen peroxide positive control (Figure 6H), indicating low acute cytotoxicity. Long-term effects on cell viability were further assessed using the Alamar Blue assay, which measures the metabolic activity of cellular oxidoreductases. As shown in Figure 6I, fraction 06 maintained approximately 63.9% and 68.6% viability at 24 and 48 h, respectively, while fraction 07 maintained 56% and 63.1% viability. These values are generally considered acceptable for non-cytotoxic compounds in a screening context. In contrast, treatment with 200 μg/mL of the crude extract reduced viability to 72.1% and 76% at 24 and 48 h, respectively, while 400 μg/mL further decreased viability to 39.3% and 21.2%—levels typically regarded as cytotoxic. These findings suggest that cytotoxic constituents present in the crude extract at high concentrations are absent from fractions 06 and 07. Collectively, these results indicate that the suppression of TNF-α expression by fractions 06 and 07 is not attributable to cytotoxic effects.

### 2.7. Fractions 06 and 07 Suppress TNF-α Expression Through Modulation of NF-κB and p38

**Figure 7:**
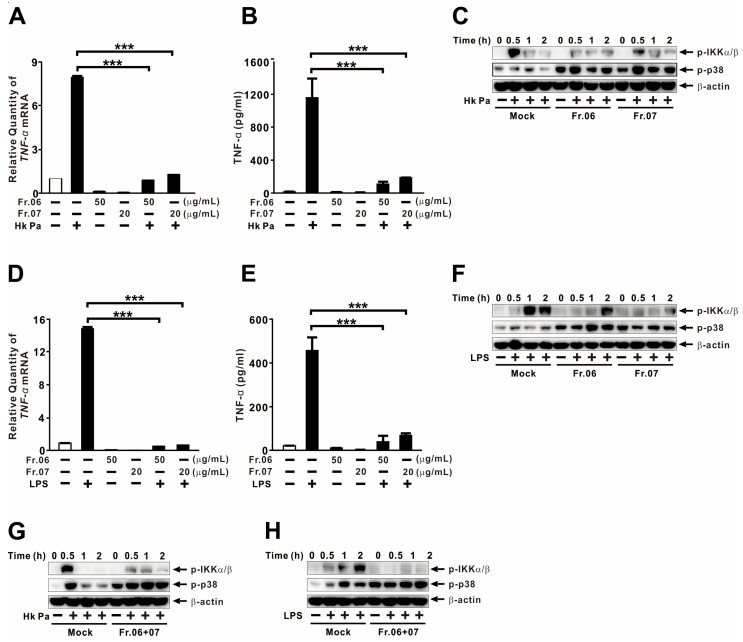
Fractions 06 and 07 suppress TNF-α expression through modulation of NF-κB and p38. THP-1 cells were pretreated with Fr.06 (50 μg/mL) or Fr.07 (20 μg/mL) for 1 h, followed by stimulation with Hk Pa (MOI 5; (**A**–**C**,**G**)) or LPS (0.5 μg/mL; (**D**–**F**,**H**)) for 4 h (**A**,**B**,**D**,**E**) or the indicated times (**C**,**F**–**H**). TNF-α mRNA levels were quantified by qRT-PCR, TNF-α protein levels were measured by ELISA, and phosphorylation levels were analyzed by immunoblotting. Data are presented as mean ± SD (n = 3). *** *p* < 0.001.

To elucidate the underlying mechanisms, we investigated whether fractions 06 and 07 modulate NF-κB and p38 signaling following stimulation with either HK-Pa or purified LPS. As shown in Figure 7A,B, pretreatment with fractions 06 and 07 significantly reduced HK-Pa–induced TNF-α expression at both the mRNA and protein levels, confirming that these fractions effectively suppress agonist-induced TNF-α production. Given that NF-κB and p38 are central regulators of TNF-α expression, we examined their involvement. Pretreatment with fractions 06 and 07 markedly reduced NF-κB phosphorylation while enhancing p38 phosphorylation (Figure 7C). Similar effects were observed under LPS stimulation: both fractions significantly decreased TNF-α expression (Figure 7D,E), accompanied by reduced NF-κB phosphorylation and enhanced p38 phosphorylation (Figure 7F). Finally, pretreatment with a mixture of fractions 06 and 07 yielded similar inhibitory results (Figure 7G,H). Taken together, these data demonstrate that fractions 06 and 07 suppress TNF-α expression by inhibiting NF-κB activation while simultaneously promoting p38 signaling.

## 3. Discussion

Inflammation is a fundamental protective response; however, the excessive production of TNF-α contributes to the pathogenesis of numerous autoimmune and chronic inflammatory disorders [46,47]. Traditional medicines have long employed botanical preparations to restore immune homeostasis, and *D. pinnata* Cav. (*Asteraceae*) has been documented in ethnomedicinal contexts for the relief of inflammatory conditions, wound healing, and related ailments. Despite these traditional applications, the biological basis of these practices has not been systematically explored. In this study, we demonstrated that *D. pinnata* extract selectively suppresses TNF-α expression in both monocytes and epithelial cells, while exerting minimal effects on IL-1β, IL-6, and IL-10 production. The extract was particularly effective in attenuating TNF-α responses to *P. aeruginosa* infection and LPS stimulation, often surpassing dexamethasone in both magnitude and rapidity. These observations suggest that *D. pinnata* contains bioactive constituents capable of selectively modulating pathological TNF-α overproduction, thereby providing a robust mechanistic rationale for its traditional anti-inflammatory use.

Mechanistic analysis revealed that TNF-α expression is largely regulated through NF-κB activation following LPS stimulation [48]. Glucocorticoids, such as dexamethasone, suppress NF-κB by upregulating IκBα and directly interfering with its transcriptional activity [49]. Consistent with this, NF-κB inhibition markedly reduced TNF-α expression; similarly, *D. pinnata* suppressed LPS-induced NF-κB activation, identifying this pathway as a primary target. NF-κB activity is further modulated by the AKT pathway, which serves to constrain excessive TNF-α production [40,50]. Although *D. pinnata* reduced AKT phosphorylation, AKT inhibition alone had negligible effects, suggesting that AKT is not essential for the extract’s suppressive activity. In contrast, PDK1 inhibition significantly suppressed TNF-α expression. Because SGK1 functions downstream of PDK1, we investigated its specific role. SGK1 exhibits context-dependent effects in TLR signaling, either suppressing inflammation [51] or enhancing cytokine production [52]. In this study, SGK1 inhibition reduced TNF-α expression; however, *D. pinnata* did not alter SGK1 activity, indicating that the observed suppression occurs independently of the SGK1 axis.

MAPK pathways are also central regulators of LPS-induced TNF-α production [53]. *D. pinnata* pretreatment enhanced ERK phosphorylation—a signaling event previously reported to suppress NF-κB-dependent gene expression by inhibiting IκB kinase [54]. However, ERK inhibition had a negligible effect in our model. More notably, we observed an increase in p38 phosphorylation alongside a reduction in TNF-α levels following treatment with *D. pinnata* extract, suggesting a negative regulatory role. While p38 MAPK is traditionally recognized as a positive regulator of TNF-α production [55,56], our findings suggest a more nuanced regulatory dynamic. It is possible that *D. pinnata* constituents influence TNF-α through p38-independent pathways or modulate post-transcriptional regulatory mechanisms. For instance, the p38 MAPK pathway, acting through the downstream kinase MK2, is known to regulate the stability of pro-inflammatory mRNAs by modulating RNA-binding proteins such as tristetraprolin [57]. Under certain conditions, p38 activation can paradoxically engage negative feedback loops that restrain cytokine production to prevent excessive inflammation. We also considered whether this suppression was mediated by IL-10, as p38 activation can enhance IL-10 production, which subsequently limits TNF-α translation [58]. In our experiments, however, *D. pinnata* extract did not affect the expression of IL-10—a potent suppressor of TNF-α [59]—suggesting that the observed suppression is not mediated through IL-10-dependent feedback. This contrasts with the mechanism of dexamethasone, which has been shown to inhibit TNF-α release in myeloid cells specifically through the inhibition of p38 signaling via the induction of MKP-1 [60]. These findings imply that *D. pinnata* and dexamethasone likely suppress TNF-α through distinct mechanisms: dexamethasone via p38 inhibition, and *D. pinnata* potentially by engaging a negative regulatory arm of the p38 pathway. Given that our data primarily demonstrate an association, further studies are required to elucidate whether p38 activation in this context serves as a compensatory feedback mechanism or a direct inhibitory signal.

Notably, the responses to dexamethasone and *D. pinnata* exhibited distinct cell-type-specific differences. While dexamethasone inhibited TNF-α in epithelial cells, it failed to suppress *P. aeruginosa*-derived LPS-induced TNF-α in monocytes under our experimental conditions. Although structural variability in the lipid A of *P. aeruginosa* LPS can alter TLR4 activation—often resulting in weaker or more selective cytokine induction [53]—we observed that dexamethasone also failed to inhibit *Escherichia coli* LPS-induced TNF-α in monocytes. This discrepancy likely reflects the time-dependent nature of dexamethasone action, as a short pretreatment period (~1 h) is typically insufficient to robustly suppress TNF-α [61,62]. Previous reports indicate that the efficacy of dexamethasone is highly dependent on incubation time; for instance, a 48 h pre-incubation may be required to reduce LPS-mediated TNF-α production to 21% of control levels. The inhibitory effect decreases significantly with shorter incubation periods: mean TNF-α production ranges from 49% to 72% of control when dexamethasone is added 8 h prior to, simultaneously with, or within 1 h after LPS stimulation. Consistently, we found that dexamethasone required prolonged exposure (≥12 h) to reduce TNF-α levels and did not significantly affect basal cytokine release in unstimulated THP-1 cells [63]. In contrast, *D. pinnata* robustly suppressed TNF-α under both basal and stimulated conditions, even with only 1 h of pretreatment, underscoring its distinct and fast-acting anti-inflammatory potential.

Bioassay-guided fractionation identified two active fractions (Fr.06 and Fr.07) that potently suppressed TNF-α expression in a dose- and time-dependent manner. In this study, these fractions exhibited higher anti-inflammatory activity at lower concentrations compared to the initial crude extract. This increase in potency is a hallmark of the fractionation process, which concentrates key secondary metabolites while eliminating bulk primary metabolites and non-specific materials. Previous studies have similarly demonstrated that solvent partitioning can substantially increase phenolic content and antioxidant capacity by refining the chemical profile [64]. Furthermore, while high concentrations of the crude extract displayed substantial cytotoxicity, Fr.06 and Fr.07 exhibited negligible acute and long-term cytotoxicity. This suggests that the fractionation process not only enriches synergistic anti-inflammatory compounds but also effectively removes toxic constituents present in the crude extract. Mechanistically, fractions 06 and 07 inhibited IKKα/β phosphorylation while enhancing p38 activation, thereby attenuating TNF-α expression in response to bacterial or LPS stimulation. These results validate the anti-inflammatory potential of *D. pinnata* and provide a foundation for the further purification and structural characterization of its active molecules.

The present study represents a significant step toward validating the ethnomedicinal use of *D. pinnata* by identifying specific active fractions and characterizing their influence on the NF-κB and p38 MAPK pathways. However, as an exploratory investigation, several distinctions must be made between these experimentally supported findings and the future research required to translate them into clinical applications. Our data provide robust in vitro evidence that bioassay-guided fractionation enriches the anti-inflammatory potency of *D. pinnata* while reducing non-specific cytotoxicity. The observed suppression of TNF-α represents a clear, mechanistically supported finding within the context of HK-*P. aeruginosa* or LPS-stimulated THP-1 cells. While UPLC-UV/MS analysis allowed for the tentative identification of several flavonoids—including apigenin-7-O-rutinoside, apigetrin, luteolin, naringenin, genistein, and isoliquiritigenin (Supplementary Materials, File S2)—which are well-known for their ability to modulate inflammatory pathways, future research must focus on preparative-scale isolation and functional characterization of these individual compounds. On the other hand, our findings are currently limited to in vitro models, which do not account for the complex pharmacokinetic profiles of a living organism. Subsequent studies using animal models of inflammation are essential to evaluate systemic efficacy and safety. Furthermore, while we have mapped the downstream signaling effects on MAPKs, the upstream molecular targets (e.g., specific cell surface receptors or intracellular binding proteins) remain to be elucidated through competitive binding assays or molecular docking studies. By clearly defining these boundaries, we affirm the pharmacological potential of *D. pinnata* while acknowledging that further rigorous investigation is required to bridge the gap between traditional knowledge and modern therapeutic standards.

## 4. Materials and Methods

### 4.1. Reagents and Inhibitors

Dexamethasone and *P. aeruginosa* strain 10-derived lipopolysaccharide (LPS) were supplied by Sigma-Aldrich (St. Louis, MO, USA). Signaling pathway inhibitors PD98059 and SB203580 were sourced from A.G. Scientific (San Diego, CA, USA); InvivoGen (San Diego, CA, USA) provided BAY 11-7082 and SP600125. Additional compounds, including GSK2334470, MK-2206, GSK650394, and CHIR99021, were obtained from Apexbio (Houston, TX, USA), MedChemExpress (Monmouth Junction, NJ, USA), Selleckchem (Houston, TX, USA), and Tocris Bioscience (Bristol, UK), respectively.

### 4.2. Preparation of Plant Extracts and Fractions

The *D. pinnata* Cav. plant extract (KPM032-014) was provided by the Natural Product Central Bank (NPCB) at the Korea Research Institute of Bioscience and Biotechnology (Cheongju, Republic of Korea). Specimens were originally harvested in 2007 from Sintaein-eup, Jeongeup-si, Jeonbuk-do, Republic of Korea. In 2024, 200 g of pulverized, shade-dried biomass was processed via ultrasonic extraction (40 kHz, 1500 W) in 2 L of HPLC-grade MeOH using an SDN-900H system (SD-Ultrasonic Co., Seoul, Republic of Korea). The protocol involved 30 sonication cycles (40 kHz, 1500 W, 15 min each) followed by a 120 min room-temperature incubation. After filtration (No. 100, Hyundai Micro Co., Seoul, Republic of Korea) and rotary evaporation, 10.25 g of crude residue was recovered. Fractionation of 4.12 g of this extract was executed on a K-PREP Lab-300 system (YMC, Kyoto, Japan) with an ODS-AQ-HG column (10 µm, 12 nm, 100 mm I.D. × 500 mm). A MeOH−water gradient (5% to 100%, *v*/*v*) yielded ten distinct fractions (Fr. 01–10), with weights ranging from 13.9 mg to 2590 mg. The yield of each fraction was as follows: Fr.01 (2590 mg), Fr.02 (142.8 mg), Fr.03 (85.3 mg), Fr.04 (173.8 mg), Fr.05 (324.7 mg), Fr.06 (265.8 mg), Fr.07 (165.3 mg), Fr.08 (75.1 mg), Fr.09 (97.0 mg), and Fr.10 (13.9 mg). All samples were reconstituted in dimethyl sulfoxide (DMSO) for bioassays.

### 4.3. UPLC-UV Profiling and Identification

The crude extract was dissolved in methanol at 3 mg/mL, and individual fractions were prepared at 1 mg/mL. An injection volume of 1 µL was used. Chromatographic analysis was conducted using a Waters ACQUITY^TM^ UPLC I-Class system equipped with a UV detector (Waters, Milford, MA, USA). An Acquity UPLC BEH C18 column (1.7 μm, 2.1 × 100 mm; Waters) was maintained at 35 °C, with the autosampler temperature set at 10 °C and a flow rate of 0.4 mL/min. The mobile phase consisted of 0.1% (*v*/*v*) formic acid in water (Phase A) and 0.1% (*v*/*v*) formic acid in acetonitrile (Phase B). Water was purified using a Milli-Q Academic system (Merck Millipore, Burlington, MA, USA); HPLC-grade acetonitrile and formic acid were purchased from Merck Millipore and Sigma-Aldrich, respectively. The gradient elution program was as follows: 0.00–1.00 min, 5% B; 1.00–20.00 min, linear 5% to 100% B; 20.00–22.50 min, 100% B; and 22.50–25.00 min, return to 5% B followed by re-equilibration. UV absorbance was monitored at 254 nm. Comprehensive UPLC-UV analysis data and the tentative identification of flavonoids are provided in the Supplementary Materials (File S1 and File S2, respectively). Within fractions Fr.06 and 07, nine major peaks were tentatively identified based on accurate precursor ion masses and their corresponding MS/MS fragment ions. Molecular formulas were proposed using high-resolution mass spectrometry (HRMS) data, supplemented by characteristic UV absorption patterns. The resulting candidates were cross-referenced with the NPCB in-house library and previously published literature.

### 4.4. Bacterial Preparation and Challenges

This study utilized *P. aeruginosa* PAK (wild-type) and its isogenic *exoSTY* deletion mutant (ΔSTY) [65]. Strains were grown in LB broth (0.5% yeast extract, 1% tryptone, and 1% NaCl, *w*/*v*) at 37 °C. Following overnight culture, cells were collected via centrifugation (20,000× *g*, 1 min) at room temperature. Pellets were either resuspended in phosphate-buffered saline (PBS) for live assays or inactivated at 65 °C for 15 min to generate heat-killed *P. aeruginosa* (HK-Pa). Experimental challenges utilized an MOI of 2 (live ΔSTY) or 5 (HK-Pa) for 4 h at 37 °C. Inflammatory priming was induced using 0.5 μg/mL LPS for 4 h.

### 4.5. Cell Line Maintenance

THP-1 (monocytes) and BEAS-2B (lung epithelial) cells were maintained at 37 °C and 5% CO_2_ in media containing 10% heat-inactivated fetal bovine serum (FBS; HyClone, Rockford, IL, USA), penicillin (100 units/mL), and streptomycin (0.1 mg/mL). THP-1 cells were cultured in Roswell Park Memorial Institute 1640 medium (RPMI 1640; HyClone), while BEAS-2B were grown in Dulbecco’s Modified Eagle’s Medium (DMEM; HyClone) supplemented with high glucose, L-glutamine, and sodium pyruvate. Cells were seeded in 6-well plates (7.0 × 10^5^ THP-1 cells/well or 4.0 × 10^5^ BEAS-2B cells/well) and maintained at 37 °C in a humidified 5% CO_2_ incubator for 16 h. Prior to treatment, cultures were transitioned to serum-free conditions for 1 h.

### 4.6. Gene Expression Analysis (qRT-PCR)

TRIzol Reagent (Invitrogen, Carlsbad, CA, USA) was employed for total RNA extraction, followed by cDNA synthesis via the ReverTra Ace qPCR RT kit (Toyobo, Osaka, Japan). Real-time PCR was performed using SYBR Green Master Mix (KAPA Biosystems, Woburn, MA, USA) on a Bio-Rad CFX96 system (Hercules, CA, USA). The primer sequences used were as follows: IL-1β, 5′-AAACAGATGAAGTGCTCCTTCCAG-3′ and 5′-TGGAGAACACCACTTGTTGCTCCA-3′; IL-6, 5′-TGGAGAACACCACTTGTTGCTCCA-3′ and 5′-GCTGCTTTCACACATGTTACTC-3′; TNF-α, 5′-CAGAGGGAAGAGTTCCCCAG-3′ and 5′-CCTTGGTCTGGTAGGAGACG-3′; IL-10, 5′-GCCTAACATGCTTCGAGATC-3′ and 5′-TGATGTCTGGGTCTTGGTTC-3′; GAPDH (internal control), 5′-CCCTCCAAAATCAAGTGG-3′ and 5′-CCATCCACAGTCTTCTGG-3′. Thermal cycling conditions consisted of 50 °C for 2 min and 95 °C for 10 min, followed by 40 cycles of 95 °C for 15 s and 60 °C for 1 min. Relative transcript levels were determined using the comparative CT method, with GAPDH serving as the internal normalization control.

### 4.7. Viability and Cytotoxicity Assays

Metabolic activity was assessed using the MAX-Blue™ Resazurin Cell Viability Assay Kit (BIOMAX, Guri, Republic of Korea) following 24 or 48 h of exposure to *D. pinnata* extract (200 or 400 μg/mL) or fractions 06 and 07 (50 or 20 μg/mL, respectively). Additionally, the CytoTox 96 kit (Promega, Madison, WI, USA) was used to measure LDH release as an indicator of cytotoxicity, following the manufacturer’s specified instructions.

### 4.8. Protein Quantification and Immunoblotting

TNF-α concentrations in supernatants were measured via DuoSet human TNF-α ELISA kits (R&D Systems, Minneapolis, MN, USA). For intracellular signaling, cells were lysed in a lysis buffer containing 20 mM Tris-HCl (pH 7.4), 50 mM NaCl, 50 mM sodium pyrophosphate, 30 mM NaF, 5 μM zinc chloride, 2 mM iodoacetic acid, and 1% Triton X-100, supplemented with 1 mM PMSF and 0.1 mM sodium orthovanadate (Sigma-Aldrich). Protein content was standardized using a BCA Protein Assay Kit (Thermo Scientific, Waltham, MA, USA). Approximately 25 μg of protein per sample was resolved by 10% SDS-PAGE and transferred to PVDF membranes. After blocking with 5% non-fat dry milk for 2 h at room temperature, membranes were probed with primary antibodies (Cell Signaling Technology, Danvers, MA, USA) against p-AKT (S473), p-IKKα/β, p-ERK, p-p38, p-JNK, p-SGK1 (S78), and β-actin (D6A8), followed by HRP-linked secondary antibodies (Cell Signaling). Protein bands were detected using enhanced chemiluminescence reagents (Thermo Scientific) and visualized with an Amersham ImageQuant-800 system (Cytiva, Marlborough, MA, USA).

### 4.9. Data Analysis

Results are expressed as mean values, with statistical significance determined by one-way ANOVA and Tukey’s post hoc test (GraphPad InStat v3.06; GraphPad Software, San Diego, CA, USA). Differences were considered significant at *p* < 0.01.

## 5. Conclusions

The present findings provide a plausible mechanistic basis for the traditional use of *D. pinnata* in managing inflammatory symptoms, such as fever and swelling. While our results offer experimental support for its anti-inflammatory potential through the modulation of the NF-κB and p38 MAPK pathways, several questions remain beyond the scope of this study. As this work was conducted entirely in vitro, these results should be interpreted as foundational. Specifically, while UPLC-UV/MS analysis allowed for the tentative identification of key peaks, these remain candidate molecules requiring large-scale isolation and definitive structural characterization via NMR. Furthermore, these in vitro observations do not account for the complex pharmacokinetics of a whole organism. Therefore, in vivo validation using inflammatory disease models is a critical next step to determine the therapeutic efficacy and safety profile of these fractions. Such studies will be crucial in determining whether the traditional ethnobotanical uses of *D. pinnata* can be translated into standardized therapeutic applications. Collectively, these results highlight *D. pinnata* as a valuable source of bioactive molecules with potential for further development, while providing an initial pharmacological validation of its ethnomedicinal applications.

## Data Availability

The original contributions presented in this study are included in the article/Supplementary Materials. Further inquiries can be directed to the corresponding author.

## Supplementary Materials

The following supporting information can be downloaded at: https://www.mdpi.com/article/10.3390/ijms27021122/s1.

## Abbreviations

*Dahlia pinnata* (*D. pinnata*); Dimethyl sulfoxide (DMSO); Enzyme-Linked Immunosorbent Assay (ELISA); Extracellular Signal-regulated Kinase (ERK); Glycogen Synthase Kinase-3 beta (GSK3β); IκB kinase alpha/beta (IKKα/β); Interleukin 1β (IL-1β); Interleukin 6 (IL-6); Interleukin 10 (IL-10); c-Jun N-terminal kinase (JNK); Lactate dehydrogenase (LDH); Lipopolysaccharide (LPS); Multiplicity of infection (MOI); Nuclear Factor kappa-light-chain-enhancer of activated B cells (NF-κB); Phosphoinositide-dependent kinase 1 (PDK1); Protein kinase B (AKT); *Pseudomonas aeruginosa* (*P. aeruginosa*); Quantitative real-time PCR (qRT-PCR); Rheumatoid arthritis (RA); Serum/glucocorticoid regulated kinase 1 (SGK1); Toll-like receptor 4 (TLR4); Triptolide (TP); Tumor necrosis factor-α (TNF-α); Type III secretion system (T3SS).
