# Supplementary material for: Traditional Medicinal Plant Dahlia pinnata Selectively Suppresses TNF-α Expression Through Modulation of NF-κB and p38 Signaling
Source: Int J Mol Sci. 2026 Jan 22;27(2):1122. doi: 10.3390/ijms27021122 (PMC12841849; doi:10.3390/ijms27021122)
Supplement: Supplementary file 1 [file ijms-27-01122-s001.zip › File S2 - Supplementary Material_Tentative ID_Fr.06 & 07.pdf]

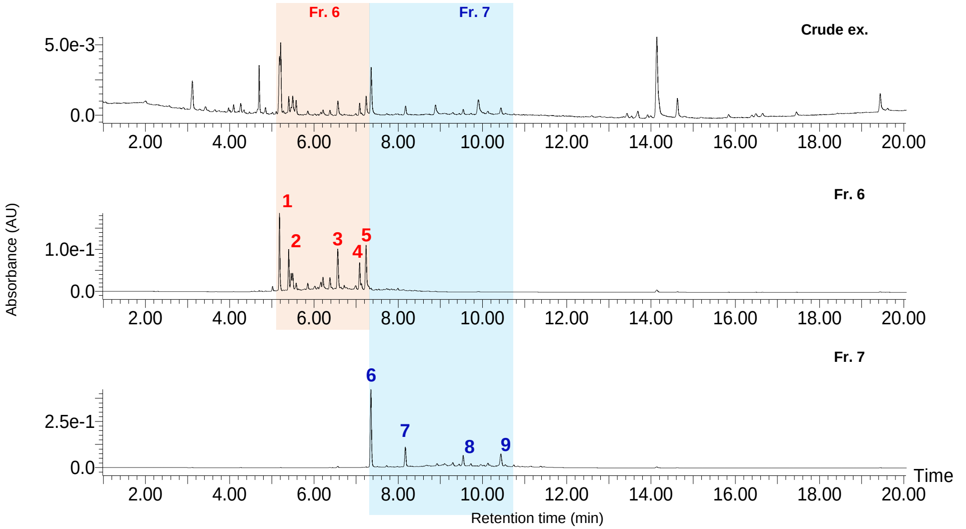

Fraction	No.	RT	Tentative ID	MF	UV	MS(-) Precusor ion [M-H]	MS(-) Calcd. ion	MS(-) Error ppm	MS(-) Fragment ions	MS(+) Precusor ion [M+H]	MS(+) Calcd. ion	MS(+) Error ppm	MS(+) Fragment ions
Fr. 6	1	5.19	Apigenin-7-*O*-rutinoside	$C_{27}H_{30}O_{14}$	266, 336	577.1552	577.1557	-0.7	269	579.1719	579.1714	0.9	271
	2	5.40	Apigetrin	$C_{21}H_{20}O_{10}$	266, 336	431.0978	431.0978	0.0	268	433.1149	433.1135	3.2	271
	3	6.56	Luteolin	$C_{15}H_{10}O_{6}$	251, 348	285.0407	285.0399	2.8	151, 133	287.0557	287.0556	0.3	153
	4	7.09	Unknown	$C_{30}H_{22}O_{10}$	280, 315	541.1138	541.1135	0.6	387,295, 159	543.1287	543.1291	-0.7	297
	5	7.23	Naringenin	$C_{15}H_{12}O_{5}$	261, 379	271.0611	271.0606	1.8	135	273.0762	273.0763	-0.4	137
Fr. 7	6	7.35	Genistein	$C_{15}H_{10}O_{5}$	266, 336	269.0452	269.045	0.7	151	271.0608	271.0606	0.7	137
	7	8.17	Isoliquiritigenin	$C_{15}H_{12}O_{4}$	370	255.0663	255.0657	2.4	119	257.0815	257.0814	0.4	353, 207
	8	9.55	Unknown	$C_{40}H_{40}O_{13}$	266, 296, 314, 335	727.2380	727.2391	-0.4	581, 329, 163, 145, 119	-	-	-	-
	9	10.45	Unknown	$C_{40}H_{40}O_{13}$	280, 297, 318	727.2395	727.2391	0.6	163, 119	751.2366 [M+Na]	751.2367	-0.1	-
