# Supplementary figures and images for: Traditional Medicinal Plant Dahlia pinnata Selectively Suppresses TNF-α Expression Through Modulation of NF-κB and p38 Signaling
Source: Int J Mol Sci. 2026 Jan 22;27(2):1122. doi: 10.3390/ijms27021122 (PMC12841849; doi:10.3390/ijms27021122)

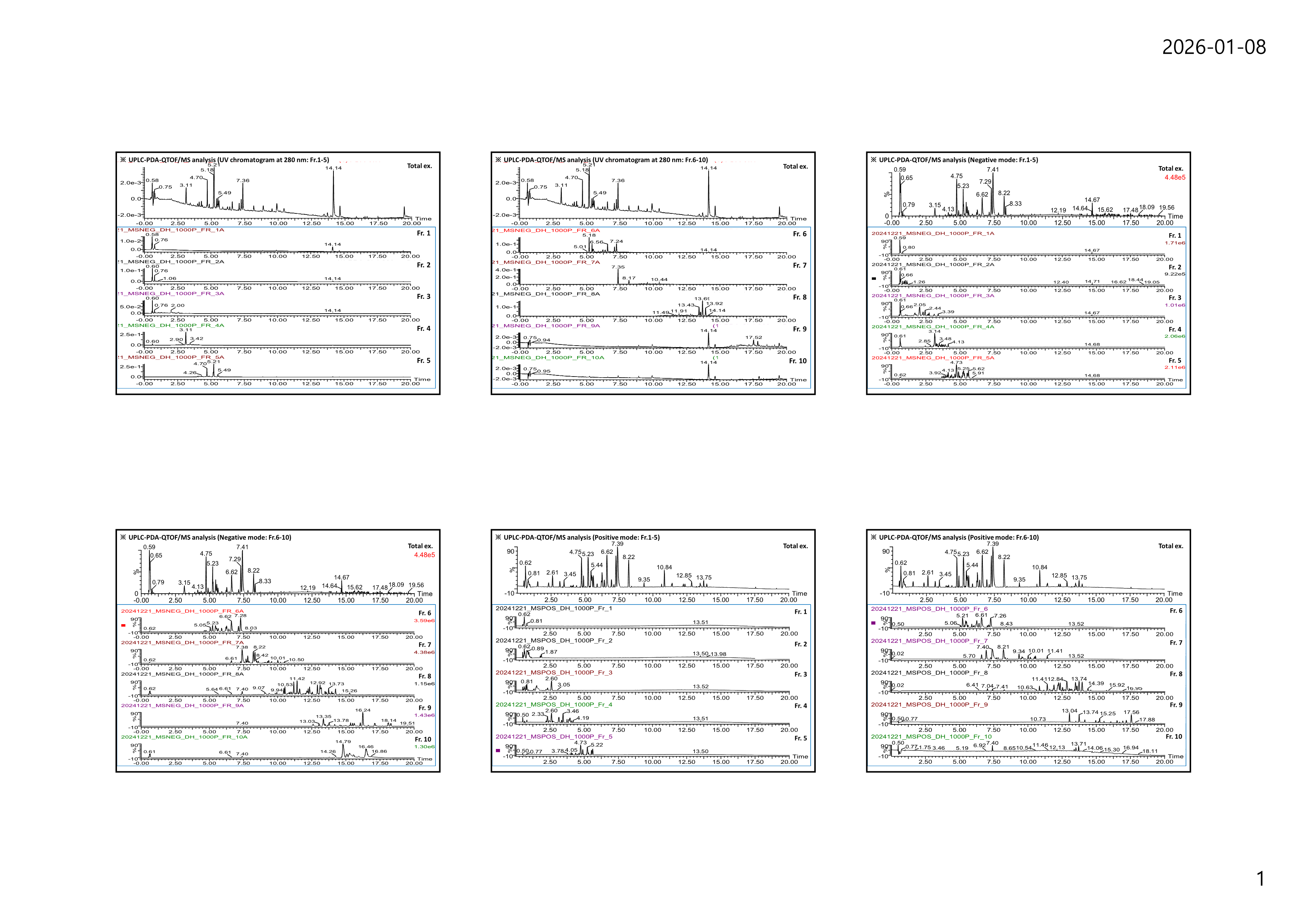

Supplement: Supplementary file 1 [file ijms-27-01122-s001.zip › File S1 - Supplementary Material_UPLC-UV analysis.tif]
